# Experimental and Modeling Study of Collagen Scaffolds with the Effects of Crosslinking and Fiber Alignment

**DOI:** 10.1155/2011/172389

**Published:** 2011-08-23

**Authors:** Bin Xu, Ming-Jay Chow, Yanhang Zhang

**Affiliations:** ^1^Department of Mechanical Engineering, Boston University, Boston, MA 02215, USA; ^2^Department of Biomedical Engineering, Boston University, Boston, MA 02215, USA

## Abstract

Collagen type I scaffolds are commonly used due to its abundance, biocompatibility, and ubiquity. Most applications require the scaffolds to operate under mechanical stresses. Therefore understanding and being able to control the structural-functional integrity of collagen scaffolds becomes crucial. Using a combined experimental and modeling approach, we studied the structure and function of Type I collagen gel with the effects of spatial fiber alignment and crosslinking. Aligned collagen scaffolds were created through the flow of magnetic particles enmeshed in collagen fibrils to mimic the anisotropy seen in native tissue. Inter- and intra- molecular crosslinking was modified chemically with Genipin to further improve the stiffness of collagen scaffolds. The anisotropic mechanical properties of collagen scaffolds were characterized using a planar biaxial tensile tester and parallel plate rheometer. The tangent stiffness from biaxial tensile test is two to three orders of magnitude higher than the storage moduli from rheological measurements. The biphasic nature of collagen gel was discussed and used to explain the mechanical behavior of collagen scaffolds under different types of mechanical tests. An anisotropic hyperelastic constitutive model was used to capture the characteristics of the stress-strain behavior exhibited by collagen scaffolds.

## 1. Introduction

Collagen, one of the major extracellular (ECM) components, is critical to the mechanical properties of many types of biological tissues including tendons, ligaments, bones, blood vessels, and skin. Collagen scaffolds have been widely used in tissue engineering, drug delivery, wound healing, and neuroregeneration guide substrate [1–3] for its biocompatibility, low toxicity, and well-documented structural, physical, chemical, and immunological properties [4]. Most of these applications require the scaffolds to operate under mechanical stresses, and thus being able to control and tailor the structural-functional integrity becomes crucial. Type I collagen gel prepared from commercially available solutions has been used broadly in biomaterials research. However, they have extremely poor biomechanical properties compared to the native tissues that they are targeted to mimic or replace. 

Collagen fibrils are strengthened by covalent crosslinks within and between the constituent collagen molecules. Aggregation of collagen fibrils forms a collagen fiber, which is the most abundant protein in the body. Collagen can self-assemble through an enzymatic formation of intermolecular crosslinks leading to a head to tail network within the fiber. The mechanical properties of collagen fibers primarily depend on the formation of intermolecular crosslinks within the fibers to prevent slippage under load [5]. However in the engineered collagen scaffolds, the density of this type of crosslinking is not large enough for practical applications. In addition to self-assembly, the overall mechanical strength of collagen fibers can be improved by increasing the density of inter- and intramolecular crosslink with various chemical reagents. 

Glutaraldehyde (GA) is one of the most common chemical crosslinking reagents for collagen [6–11]. Collagen gels crosslinked with GA have already been studied for ocular surfaces [12], corneal tissue engineering scaffolds [4], and nanoscale collagen fibril scaffolds [8, 13]. GA helps to retain many of the viscoelastic properties of collagen fibrillar network, and it reacts relatively quickly. Addition of GA will induce covalent bonds between collagen fibrils from aldehyde-amino reactions as well as from aldol condensation [14]. This results in a more tightly crosslinked network. GA can also lead to intramolecular crosslinks formed between two *α*-chains by aldol condensation. Although widely used as a crosslinking reagent for collagen-based biomaterials, the cytotoxicity problem associated with GA is a recognized draw back and prevents its application from *in vivo* studies.

Recently genipin (GP), a compound extracted from the fruit of the Gardenia Jasminoides, has been shown to effectively crosslink cellular and acellular biological tissues as well as many biomaterials including hydrogels and hydrogel composites [7]. It was also found that GP is significantly less cytotoxic than GA [15, 16]. Such features make GP an alternative crosslinking agent for biomaterials with improved mechanical properties. Similar to GA, GP can also form intramolecular as well as intermolecular crosslinks in collagen [7]. GP spontaneously reacts with the primary amines, lysine and arginine residues, in collagen to form monomers that further crosslink the collagen [17]. However, the crosslinking procedure is a complex process, and little is known about the mechanical properties of collagen treated with GP. 

Preferred collagen orientation along the dominant physiological loading direction has been observed in many previous studies [18, 19]. Mechanical anisotropy in native tissue is highly associated with fiber orientation. Assembly of collagen molecules *in vitro* remains a major challenge for fabricating the next generation of engineered tissues. There are several ways to achieve anisotropic-aligned collagen fibrils during assembly. Molecules can be aligned by flow, microfluidic channels [20] and the application of external anisotropic mechanical forces [21–23], electric currents [24], and magnetic fields. Constant magnetic fields are able to align collagen molecules because the collagen molecules have diamagnetic anisotropy. Barocas et al. [25] demonstrated the circumferential alignment of the collagen in a tubular mold. However, the small diamagnetism of collagen molecules requires Tesla-order strengths magnet [26]. Recently, Guo and Kaufman [27] utilized the flow of magnetic beads enmeshed in collagen fibrils to align collagen. The streptavidin-coated ferromagnetic beads (about 1.5 *μ*m in diameter) were shown to facilitate collagen alignment under magnetic field as low as 10^−4^ T. Collagen was aligned as the beads move towards the magnetic poles. The timescales of beads travel and gelation need to be comparable for the alignment to occur properly. 

As a network with hierarchical structures, the relationship between the mechanical properties of collagen and its structures is obviously causal. The present study was designed to characterize the biaxial tensile and rheological mechanical properties of collagen scaffolds. Different concentrations of GP were used to modify the degree of crosslinking in the collagen scaffolds. The method of using the flow of magnetic beads enmeshed in collagen fibrils [27] was adapted to achieve the alignment of fibers in the scaffolds. The coupled effects of fiber alignment and crosslinking across hierarchies on the mechanical properties of collagen scaffolds were studied. 

## 2. Materials and Methods

### 2.1. Sample Preparation

**Genipin Crosslinked Collagen Scaffolds**

**Figure 1:**
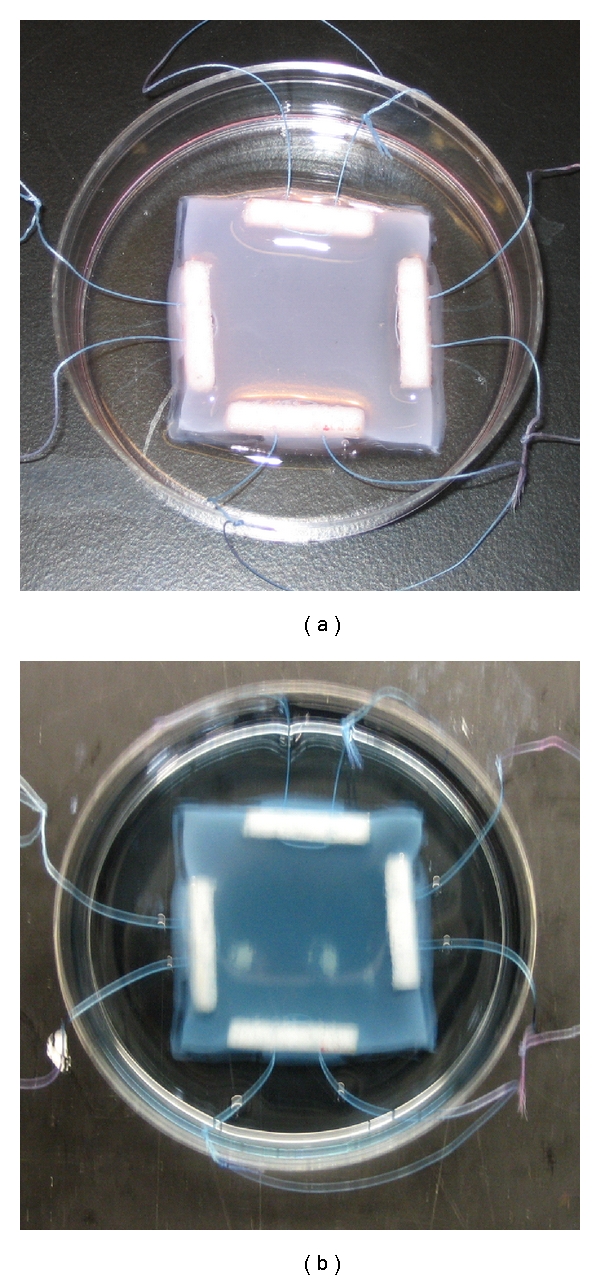
Collagen scaffolds crosslinked with (a) 0.03% and (b) 0.1% GP for 6 hours. Four prethreaded polyethylene bars were polymerized into the collagen scaffolds sample for biaxial tensile testing.

Nutragen type I collagen solution (6 mg/mL) was purchased from Advanced BioMatirx. Collagen was dissolved in 0.01 N HCl with a pH value of approximately 2.0. Neutralized collagen solution was prepared by quickly mixing Nutragen collagen solution, 10x PBS (Fisher Scientific), and 0.1 M NaOH (Fisher Scientific) solution with a ratio of 8 : 1 : 1 at 4°C with a final collagen concentration of 4.8 mg/mL. The pH value of the solution was adjusted to be between 7.2~7.4. The neutralized solution was transferred into a custom-made square reservoir with a dimension of about 30 mm × 30 mm that sits in a Petri dish. On each side of the reservoir, a 15 mm  × 1 mm notch was cut to fit the loading bars. Four porous polyethylene bars (18 mm × 3 mm × 1.5 mm) (Fisher Scientific) prethreaded with nylon sutures were placed by the sides of the reservoir. The dimension between polyethylene bars was about 15 mm × 15 mm, and the thickness of the gel was about 1 mm. The solution was firstly kept in an incubator at 37°C for 12 hours for gelation [4, 6, 11]. During gelation, the polyethylene bars were polymerized into the collagen gels [28] (Figure 1). The collagen gels were then immersed in 0.03%, 0.1%, and 0.25% GP solutions for another 6 hours in the incubator for crosslinking [16].

**Magnetically Aligned Collagen Gel**

The aligned collagen scaffolds were obtained with the aid of a flow of magnetic particles embedded in collagen fibrils [27]. Briefly, neutralized collagen solutions were prepared as above. Streptavidin-coated iron oxide magnetic particles (Bangs Labs) of 1.5 *μ*m in diameter were added into the neutralized collagen solution at a concentration of 0.1 mg/mL. The solution with beads was then transferred into the reservoir and incubated for 12 hours at 37°C, during which a magnetic bar was placed under the petri dish. The direction of the magnetic field was marked on the petri dish. After gelation, samples were immersed in 0.03%, 0.1%, and 0.25% GP solutions for further crosslinking.

### 2.2. Scanning Electron Microscopy (SEM)

The morphology of the collagen scaffolds was examined using a JOEL JSM-6100 SEM operated at 10 kV. To prepare the sample for SEM, crosslinked collagen gels were fixed with 4% paraformaldehyde in PBS for 1 hour at room temperature. The fixed samples were dehydrated in a graded distilled water/ethanol series: 30%, 50%, 70%, and 100% for 15 minutes each, followed by washes with a graded ethanol/HMDS series: 30%, 50%, 70%, and 100% for 15 minutes each, and finally allowed to dry overnight [29–31]. This drying process has been employed to avoid sample shrinkage. The dried hydrogels were sputter-coated with Pd/Au prior to SEM. SEM images were taken at multiple locations across the sample and used to qualitatively assess the alignment of collagen fibers.

### 2.3. Planar Biaxial Tensile Test

The tensile mechanical properties of collagen scaffolds were characterized using a planar biaxial tensile tester. In biaxial tensile testing, a roughly square-shaped specimen was mounted so that it could be stretched along both the *x* and *y* in-plane directions. Four carbon dots markers forming a 5 mm × 5 mm square were placed in the center of the testing specimen, and a CCD camera was used to track the position of markers from which the tissue strains in both directions can be determined throughout the deformation. Tensile tension was applied to the specimen and the load was measured using load cells during the loading and unloading processes. The square samples were loaded biaxially via sutures prethreaded to the polyethylene bars. A preload of 2 g was used to straighten the sutures. Samples were preconditioned equibiaxially for 8 cycles with a load of 10 g to achieve a repeatable material response. A half cycle time of 10 seconds was used. The preloaded state was used as the reference state for later strain calculation. The samples were then tested under load control method and subjected to a set of equibiaxial loads with the maximum loads varying from 70 g to 100 g. Cauchy stress and logarithm strain were calculated [32] and used for the description of the biaxial tensile mechanical behavior of collagen gel.

### 2.4. Rheometry Study

The mechanical properties of collagen gel were also assessed using a parallel plate rheometer (AR2000, TA Instrument). Neutralized collagen solution was prepared as stated above with a final collagen concentration of 4.8 mg/mL. The solution was transferred to P60 Petri dishes and incubated at 37°C for 12 hours. The collagen gels were then crosslinked with 0.03%, 0.1%, and 0.25% GP for 6 hours. Before rheometry tests, the gel samples of 60 mm in diameter and 2 mm in thickness were carefully removed from the Petri dish and transferred to the bottom plate of the rheometer. The temperature of the plate was set to 37°C. The top plate was lowered to a height of 0.9 mm. Frequency and strain sweep tests were performed. For frequency sweep test, the dynamic storage and loss moduli were evaluated at 1% shear strain amplitude at frequencies ranging from 0.1 to 10 Hz. For strain sweep test, the moduli were evaluated at shear strain ranging from 0 to about 10% at 5 Hz. 

### 2.5. Constitutive Modeling

In order to capture the characteristics of the stress-strain behavior exhibited by collagen gels, an anisotropic hyperelastic constitutive model was used [33]. The strain energy function was originally introduced by Holzapfel et al. [34] for the description of the passive mechanical response of arterial tissue, in which each layer is treated as a fiber-reinforced material with the fibers corresponding to the collagenous component distributed helically around the arterial wall. The model was later generalized to include collagen fiber dispersion [33]. The structurally based constitutive model has material parameters possessing physical meanings that can be related to the structure and components of the material being studied. The constitutive model was chosen in this study as it has been successfully used in previous studies for collagenous cardiovascular tissue and elastin-degraded arteries [35–37]. The general form of the strain energy function is

(1) $$\begin{array}{cc} U & =C_{10}\left({\overset{̅}{I}}_{1}-3\right)+\frac{k_{1}}{2k_{2}}\overset{N}{\underset{\alpha =1}{\sum }}\left\{\exp\;\left[k_{2}{\langle {\bar{E}}_{\alpha }\rangle }^{2}\right]-1\right\}\\ & \;+\frac{1}{D}\left(\frac{{\left(J^{\text{el}}\right)}^{2}-1}{2}-\ln\;J^{\text{el}}\right). \end{array}$$

**Figure 2:**
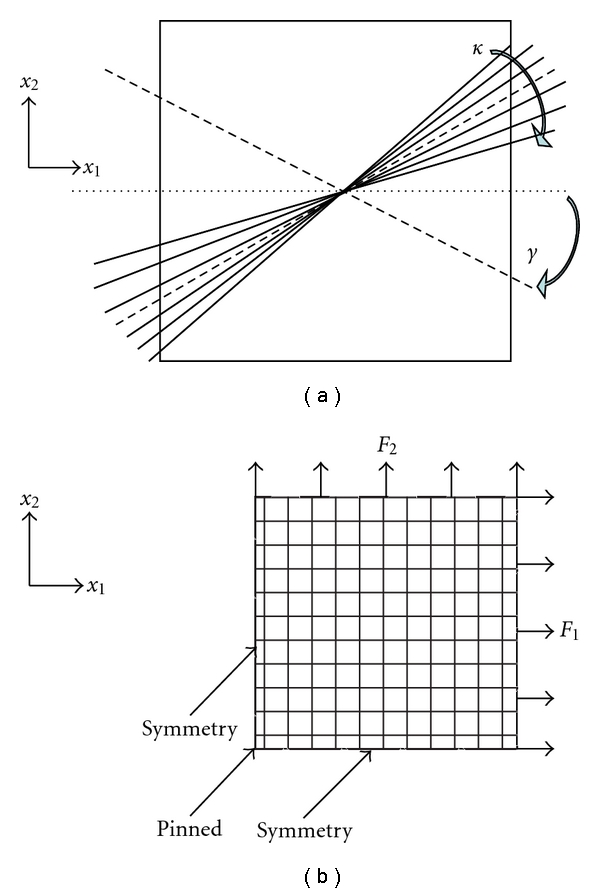
(a) Definition of fiber orientation *γ* and fiber dispersion *κ* in a tissue sample. (b) Finite element model with loading and boundary conditions.

In (1), ${\bar{E}}_{\alpha }\overset{\mathrm{def}}{=}\kappa \left({\bar{I}}_{1}-3\right)+\left(1-3\kappa \right)\left({\bar{I}}_{4(\alpha \alpha )}-1\right)$, where *κ* = (1/4)∫_0_^*π*^ *ρ*(Θ)sin^3^Θ*d*Θ. The response of the model is governed by two parts, the isotropic and anisotropic components. The first term in (1) captures the isotropic behavior of the matrix material. ${\bar{I}}_{1}$ is the first invariant of tensor $\bar{C}$, where $\bar{C}\;$is the modified counterparts of right Cauchy-Green tensor [34]. *C*_10_  is a stress-like material parameter associated with the isotropic part of the overall response of the tissue. The anisotropy of the tissue is captured by the second term in (1). An assumption in this model is that the collagen fibers are only active in extension and not in compression. The 〈⋯〉 stands for the Macauley bracket and imposes the condition that$\;{\bar{E}}_{\alpha }>0$. *k*_1_ is another stress-like parameter, and *k*_2_ is a dimensionless constant related to the collagen fibers. *N* is the number of families of fibers, and *κ* is a measure of the dispersion of the fiber orientation around a mean direction, as shown in Figure 2(a). The mean direction of the fibers, *γ*, is defined as the angle between the circumference direction of the tissue and the mean direction of the fibers. *ρ*(Θ) is the orientation density function and gives the normalized number of fibers within a certain orientation with respect to the mean direction. ${\bar{I}}_{4(\alpha \alpha )}$ are squares of the stretches in the direction of the *α* family of fibers. Finally, *D* is the inverse of the bulk modulus and is set to zero as the material is assumed to be incompressible. $J^{\text{el}}={\left(\det\;\bar{C}\right)}^{1/2}$ is the elastic volume ratio. Note that *J* = 1 for incompressible material. Interested readers are referred to Gasser et al. [33] for more detailed explanations of the model.

Due to the symmetric loading conditions in biaxial tensile test, a quarter of the collagen sample was modeled in ABAQUS 6.8-4 with loading and boundary conditions shown in Figure 2(b). Shell edge loading *X*- and *Y*-symmetry boundary conditions were applied in order to simulate biaxial tensile testing experimental settings. General-purpose shell elements (S4R) with inherent plane stress assumption were used in the finite element model. Material parameters were adjusted to fit the simulation results to experimental stress-strain curves.

## 3. Results

The color appearance of collagen gels changes when crosslinked with crosslinking reagents. Thermally crosslinked collagen gels at 37°C without any crosslinking reagents have a whitish color and are semitransparent. These scaffolds are extremely fragile and cannot be tested mechanically. Collagen gels cross-linked with GP turn into bluish color and become opaque, as shown in Figure 1. The higher concentration of GP increases the intensity of blue. 

**Figure 3:**
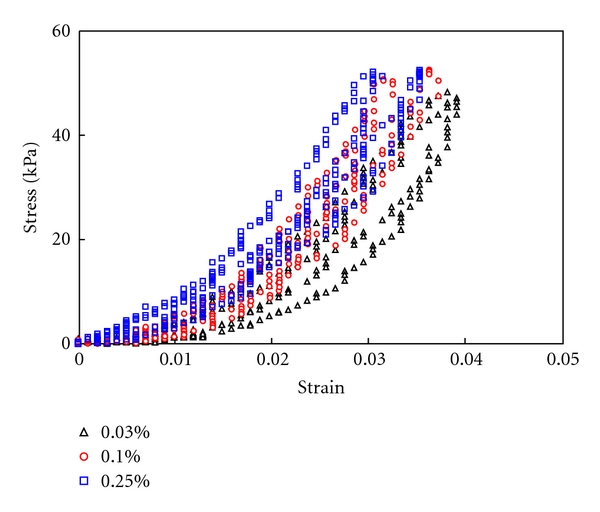
Representative stress-strain responses of collagen scaffolds crosslinked with 0.03%, 0.1%, and 0.25% GP subjected to equibiaxial tensile test. The maximum load is 70 g, 100 g, and 100 g for 0.03%, 0.1%, and 0.25% GP crosslinked samples, respectively.

**Figure 4:**
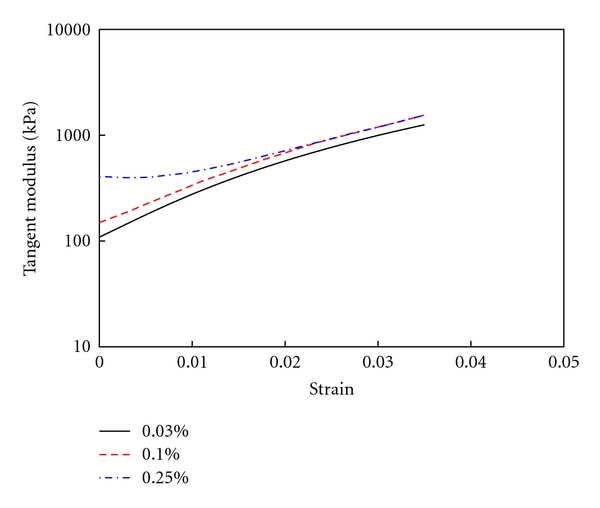
Average tangent modulus of the collagen scaffolds with 0.03%, 0.1%, and 0.25% GP.

Figure 3 shows the representative stress-strain responses of collagen scaffolds crosslinked with different concentration of GP. Samples were under equibiaxial tensile test with a maximum load of 70–100 g. The biaxial tensile test revealed that the stiffness of collagen gel increases with higher concentrations of GP. It is also noted that all collagen scaffolds exhibit isotropic mechanical behavior as seen from the equibiaxial testing results. To further compare the stiffness of crosslinked collagen gels, the tangent modulus *E*_*t*_ was obtained for each sample by differentiating the stress-strain curves and was estimated from *E*_*t*_ ≈ 0.5 × *dσ*/*dε* [38]. Averaged tangent moduli were then obtained for collagen scaffolds with 0.03%, 0.1%, and 0.25% GP.  Figure 4 shows that the initial tangent moduli of collagen scaffold increase with the concentration of GP. The tangent moduli also increase with strain. For strain less than 2%, the tangent moduli of the 0.03% and 0.1% GP crosslinked collagen scaffolds increase faster than the 0.25% GP crosslinked one. However, the tangent modulus of the 0.25% GP crosslinked collagen gel remained the highest. When strain is higher than 2%, the tangent moduli of the 0.1% GP crosslinked scaffolds are about the same as the 0.25% GP crosslinked scaffolds. As the strain further increases, the tangent moduli increase at about the same rate for all the samples. 

**Figure 5:**
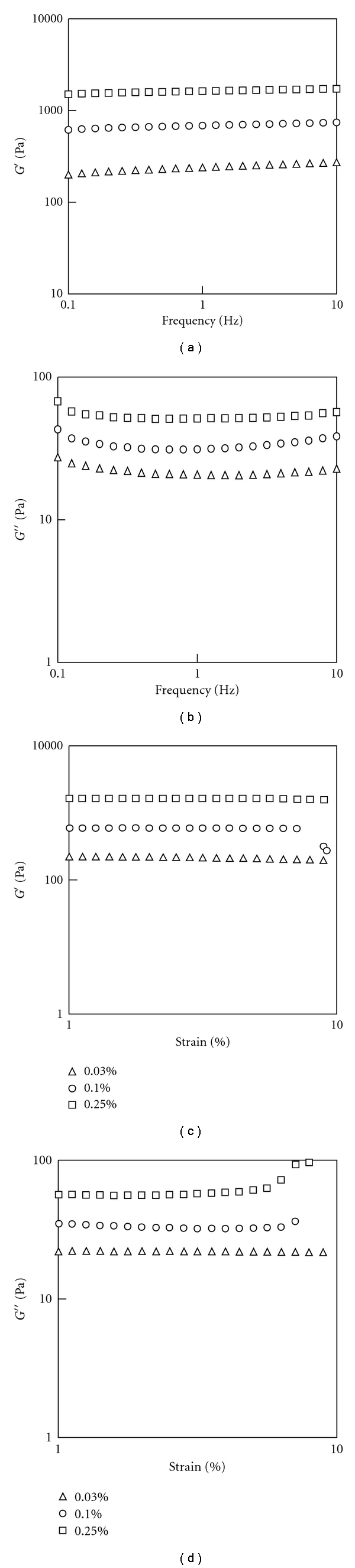
(a, b) Dynamic storage and loss moduli from frequency sweep rheological tests; (c, d) from strain sweep rheological tests of collagen scaffolds crosslinked with 0.03%, 0.1%, and 0.25% GP.

Rheological testing with a parallel plate rheometer demonstrate that the storage (*G*′) and loss (*G*′′) moduli of GP-crosslinked collagen scaffolds both increase with GP concentration, as shown in Figures 5(a) and 5(b). The *G*′ increases slightly with frequency, while the *G*′′ decreases with frequency initially followed by a slight increase. The *G*′ of collagen gels is about 10 times greater than the *G*′′ which suggests that collagen gel is a predominantly elastic material with small viscosity. The variation of storage and loss modulus with shear strain amplitudes are presented in Figures 5(c) and 5(d). There is a slight decrease in the storage moduli and an increase in the loss moduli with increasing strain amplitude. Overall, the storage and shear moduli do not vary significantly within the shear strain range applied in this study. 

**Figure 6:**
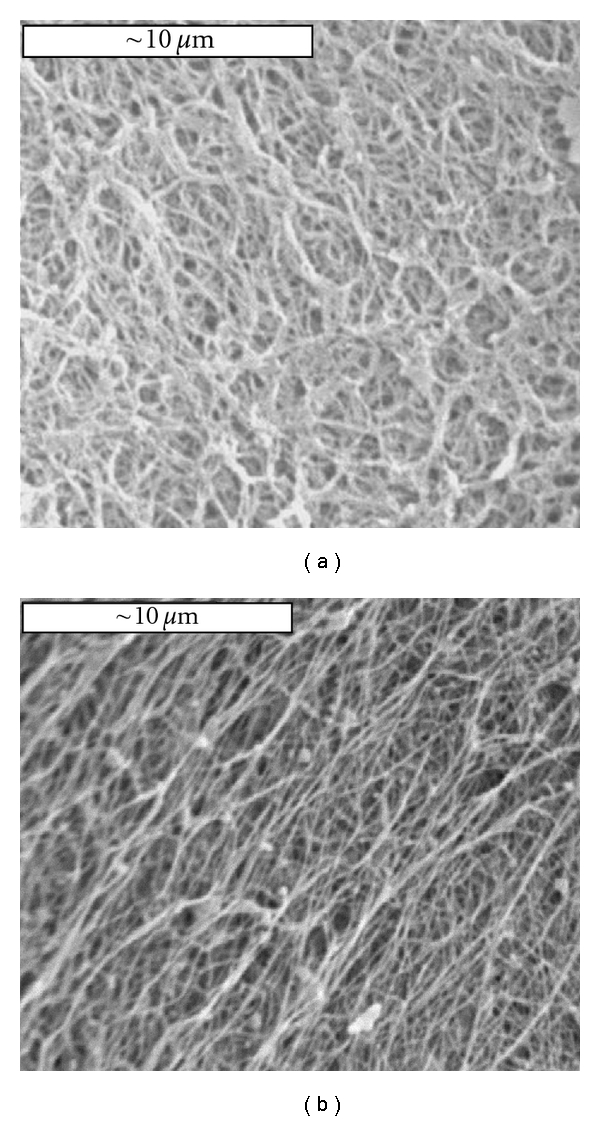
Scanning electron microscopy (SEM) images of (a) nonaligned and (b) aligned 0.03% GP crosslinked collagen scaffolds. All the scale bars represent 10 *μ*m.

**Figure 7:**
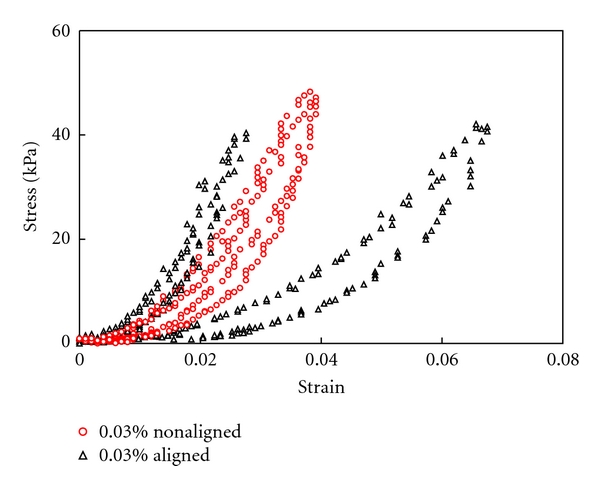
Representative stress-strain response of the aligned and non-aligned collagen scaffolds crosslinked with 0.03% GP. Equi-biaxial tensile test reveals obvious anisotropic mechanical behavior in the aligned collagen scaffold.

SEM was performed to examine the structure in the aligned collagen scaffolds. The nonaligned collagen gel was also examined for comparison. As shown in Figure 6(a), the fibers in the nonaligned collagen gel distribute randomly and there is no preferred fiber distribution. On the other hand, the fibers in the aligned collagen gel show an overall fiber alignment in a particular direction, as shown in Figure 6(b). The mechanical behavior of the aligned collagen scaffolds were tested using a biaxial tensile tester and compared with the results from the nonaligned ones. The samples were subjected to equibiaxial tensile test with one loading axis parallel to the fiber alignment direction while the other loading direction perpendicular to the fiber alignment direction. The nonaligned collagen scaffolds with magnetic beads imbedded were also tested to validate that the presence of beads would not affect the mechanical property of collagen scaffolds (results now shown). The stress-strain responses of the aligned collagen scaffolds are shown in Figure 7. Compared with the nonaligned scaffolds, the aligned ones demonstrate obvious anisotropic mechanical behavior as manifested by one direction being stiffer than the other. As expected, the scaffolds are stiffer in the direction parallel to fiber alignment than in the direction perpendicular to the fiber alignment. The nonaligned collagen scaffolds appear to be isotropic with the stress-strain curves falling in between those from the aligned scaffolds.

**Figure 8:**
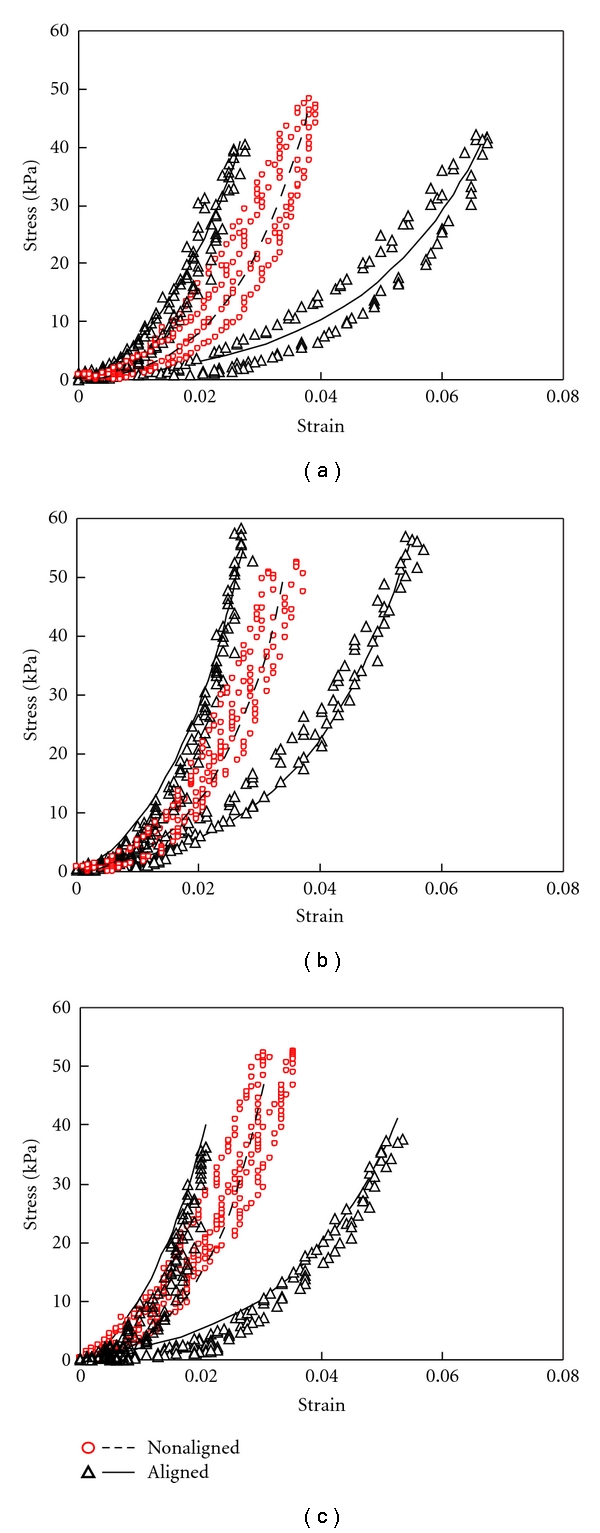
Simulation results of Cauchy stress versus logarithmic strain for isotropic and anisotropic collagen gels crosslinked with (a) 0.03%; (b) 0.1%; (c) 0.25% GP. Isotropic fits are shown with the dotted lines. Anisotropic fits are shown with solid lines. Experimental results are shown with the symbols.

Figure 8 shows the simulation results. Experimental results are also plotted for comparison. For nonaligned collagen scaffolds, *γ* was set to 45° and *κ* was set to 0.333 to represent an isotropic material. The *C*_10_, *k*_1_, and *k*_2_ values were then chosen in order to fit the model to experimental data. For the aligned scaffolds, *C*_10_ was kept the same as the corresponding nonaligned one and appropriate choices of *k*_1_, *k*_2_, *γ*, and *κ* were made to fit the experimental data. The material parameters for the models are summarized in Table 1. 

## 4. Discussion

### 4.1. Effect of Crosslinking

The change of color in collagen scaffolds crosslinked with chemical reagents indicates microstructure changes during crosslinking. For GP crosslinked collagen, blue pigment is produced by the reaction between collagen and GP [39]. It was proposed that the reaction between GP and an amino acid in collagen molecule will form a monomer and further radical reaction will cause dimerization. The mixtures of polymers formed from these reactions are the cause of blue pigment [40]. Previous studies suggested that GP may form intramolecular and intermolecular crosslinks within collagen fibers in biological tissue [7].

Various methods have been developed to improve the mechanical properties of collagen scaffolds including physical crosslinking through exposure to a radiation sources such as UV light [41, 42] and chemical crosslinking by chemical reactions with various crosslinking reagents [6, 43]. Glutaraldehyde is commonly used for collagen-based biomaterials. It has been shown that the stiffness of collagen gel increases with higher GA concentration [11]. However, this trend stops when the GA concentration is higher than a threshold value. Previous study using dermal sheep collagen [6] showed that collagen network treated with 0.08% and 0.5% GA had a similar modulus. Sheu et al. [11] suggested that a complete crosslinking in collagen gel is reached when the GA concentration is 0.12%. After fully crosslinking, no more amino groups will be available to react with the aldehydes from GA. Lacking of amino groups in the collagen gel will lead to self-polymerization of GA, which may adversely affect the mechanical property of the entire collagen gel [9, 14]. Although GA crosslinking greatly improved the mechanical strength of collagen gel, the potential toxic effect has been a vital drawback of this commonly used chemical reagent for biological tissues. A few studies have demonstrated that GP has the potential to be used as a substitute crosslinking reagent [7, 15, 16, 44]. Among these studies, GP has been found to be significantly less cytotoxic than GA [15, 44]. From the mechanical perspective, previous studies found that GP can be used to stiffen collagen gels in a relatively short-time frame. It is also noted that the mechanical properties of collagen gel can be controlled by varying the concentration of GP. Sundararaghavan et al. [16] found that the degree of crosslinking and the storage modulus of GP-crosslinked collagen gel increased with higher GP concentration. Our results from both biaxial tensile testing (Figures 3 and 4) and rheometry measurements (Figure 5) show that the tangent modulus, the storage, and loss modulus of collagen scaffolds increase with GP concentration. Although genipin can be used to tune the overall stiffness of collagen scaffolds, higher concentrations of genipin can lead to significant cell death [16], which needs to be considered in applications of cell-populated collagen scaffolds.

### 4.2. Effect of Fiber Alignment

Mechanical properties of collagen scaffolds are not only affected by the degree of crosslinking, but also highly correlated with the orientation of collagen fibers in the scaffolds. Preferred fiber orientation along the dominant physiological loading direction has been observed in many previous studies on various tissues [45–47]. Collagen fiber orientation plays important roles in determining the mechanical functionalities of collagen-based native and engineered biological material. In the present study, the alignment of collagen fibers was achieved via the flow of streptavidin-coated magnetic beads bond with collagen fibrils proposed previously by Guo and Kaufman [27], to which interested readers are referred for greater detail. Briefly, the streptavidin coating contains an Arg-Tyr-Asp (RYD) amino acid sequence similar to the RGD receptor domain of fibronectin, which has a function of binding with collagen fibrils. Our results demonstrated that, with this simple technique, the resulted scaffolds possess obvious anisotropic mechanical properties as manifested by the stress-strain responses from equibiaxial tensile test (Figure 7). Such kind of preliminary results are usefully for future quantitative investigation of the structure-function relation of collagen scaffolds. The anisotropic scaffolds are stiffer in the direction that the fibers are aligned than in the direction perpendicular to the alignment. 

Our results also demonstrated that crosslinking of the scaffolds can be used to tune the overall stiffness of the scaffolds without affecting the existence of anisotropy in the collagen gel. Previous study by Sung et al. [7] found that fixation of the aortic valves using GP and GA did not alter the mechanical anisotropy observed in fresh valve leaflets. They concluded that the intramolecular and intermolecular crosslinks introduced into the collagen fibrils during fixation are of secondary importance to the presence of structural and mechanical anisotropy in fresh leaflets. Paik et al. [48] studied the effect of nitrite on the mechanical properties of uniaxially and biaxially constrained collagen gels. They quantitatively demonstrated that the crosslinking agent did not alter the fiber distribution in collagen gels significantly. Their study also suggested that the stiffening of collagen fibers might be another source of the anisotropic mechanical behavior seen in the uniaxially constrained collagen gel, in addition to the reorientation of fibers in the constraint direction. In the present study, the reorientation of collagen fibers induced by magnetic flow plays a dominant role in controlling the anisotropic mechanical behavior of collagen scaffolds. 

### 4.3. Comparison between Biaxial Tensile and Rheological Testing

Planar biaxial tensile testing was used in this study to characterize the tensile mechanical properties of collagen scaffolds. Planar biaxial tensile test with independent control of load in both perpendicular loading directions has been used broadly to study the mechanical behavior of various native and engineered biological tissues [28, 32, 49, 50]. Although it cannot replicate the physiological loading conditions, biaxial tensile test is sufficient on elucidating the anisotropic mechanical properties of biological tissues with plane stress assumptions. In the present study, equibiaxial tensile testing was performed to study the effects of crosslinking and fiber-alignment on the mechanical properties of collagen scaffolds. Rheological testing has been applied to study the mechanical properties of polymeric and biological materials [29, 51, 52]. Our results show that higher GP concentration will generate a collagen gel with higher dynamic storage and loss moduli, which is consistent with previous study by Sundararaghavan et al. [16]. Although biaxial tensile testing results show a similar trend, the tangent stiffness from biaxial tensile test is two to three orders of magnitude higher than the storage moduli from rheological measurements, which is much greater than 3 : 1 for ideal elastic networks. Higher ratio of tangent modulus to shear modulus has been observed in previous studies on biomaterials and hydrogel system. Using a three-point bending test, Spatz et al. [53] reported that the ratio of Young's modulus to shear modulus of cortical bone is in the order of 20 : 1. They suggested that materials comprising stiff fillers embedded in a compliant matrix could have a rather low shear modulus. Such kind of properties allow hollow bones to react smoothly to local impacts, which otherwise may lead to failure. Study by Richter [54] on poly(vinyl methylether) hydrogels showed that the ratio of compressive to shear moduli varied from 4.8 to 10.9 [54], which was contributed to the inhomogeneity of the sample. 

It is known that collagen gel is a biphasic system consisting of a fibrillar network structure filled with a large excess of interstitial fluid. Tensile tests on collagen gels probe the tensile behavior of the fibrillar network, and the interstitial flow resistance is negligible in extension tests [55]. Results from our biaxial tensile testing on collagen scaffolds give a nonlinear stress-strain response with a compliant “toe” region, followed by a stiff region. The initial compliant “toe” region is due to the network orientation changes toward the loading axis, while the stiff region corresponds to resistance from fibril extension [56]. In rheological test, the fluids or soft solids flow rather than deform elastically [57]. The top plate of the rheometer compresses the collagen gel, oscillates, and exerts a dynamic torsional force on the sample. During the unconfined compression fluid, escape may not be trivial anymore. Another reason which may contribute to the low-measured shear modulus is that, during rheological test, fluid running out of the collagen gel can act as a lubricant between the gel surface and top plate. Thus the gel might in fact experience smaller shear than that applied by the top plate. This lubricant effect may dominate the increase in storage and loss moduli associated with dehydration. Results from our study suggest that, in rheological test, the movement of the interstitial fluid play a significant role in determining the overall mechanical properties of a collagen gel. It is important to understand the different mechanical properties that a biphasic collagen gel can exhibit under specific loading conditions, as such understandings are necessary for mechanobiological studies in which ECM mechanics play critical roles.

### 4.4. Determination of Material Parameters in the Constitutive Model

The anisotropic constitutive model in (1) was proposed originally for simulating arterial tissue in which elastin is the primary means of support at low stress-loading regions and then collagen being the major load-bearing component at higher stresses [33, 34]. In an earlier study by Holzapfel et al. [58], individual layers of arteries were separately modeled with their own sets of parameters. Their study using the constitutive model by Holzapfel et al. [34] was successful in modeling the intimal and adventitial layers that lack two distinct structural components as the medial layer. Regarding the parameters in the model with respect to our collagen gels, there was no elastin or other ECM ground substance material that support the initial loading in this experiment. The *C*_10_ parameter is likely more representative of some other phenomenon such as uncoiling of collagen fibers. Parameters *γ* and *κ* can vary the amount of anisotropy in the stress-strain curves, and *κ* has a greater effect on the amount of curvature in the stress-strain curve [33, 59]. Finally, the most significant effect of increasing/decreasing *k*_1_ and *k*_2_ is in translating the stress-strain curves to lower/higher strains, respectively. This inverse relationship in the material is logical as they describe the properties of the collagen fibers [58]. 

Examining the parameters for the aligned and nonaligned collagen gels, we initially attempted to take the parameters from the nonaligned simulations and only change *γ* and *κ* to create a best fit of the aligned collagen gel, since *γ* controls the orientation of the fibers, and *κ* determines the fiber dispersion (amount of fiber alignment). Fitting of *γ* and *κ* has generally been done phenomenologically instead of based on histology studies [33]. However, it is interesting to point out that our results indicate a correlation between the degree of anisotropy demonstrated by stress-strain curves and *γ*. Among the three aligned collagen scaffolds shown in Figure 8, the collagen scaffolds aligned with 0.03% GP possessing the highest amount of anisotropy has the smallest model parameter *γ* correspondingly. Unfortunately, a good fit of the aligned collagen gel was not possible without modification of the *k*_1_ and *k*_2_ as well. This seems to indicate that the alignment process is affecting the material properties of the collagen fibers and not just their orientation/dispersion. 

In the nonaligned and aligned collagen scaffolds, *C*_10_ values were increased with higher GP concentrations, which suggest higher *C*_10_ values for stiffer tissues resulted from increased crosslinking. There was also an increase in the values of *k*_1_ and *k*_2_ with GP concentration. Previous studies have shown that the method of crosslinking can affect how the crosslinks are organized in the collagen fibers [60]. Genipin has been shown to not only cause collagen to become more crosslinked but also cause the fibers to merge together and form thicker structures [61]. Increase in *k*_1_ and *k*_2_ indicates that stronger fibers present in collagen gels with higher GP concentration. Takiuchi et al. [62] showed that crosslinking along fibers may happen more often than across fibers. They showed that during the process of fixing tendon samples in formalin, the stiffness along the fibers increased linearly with fixing time. However, this is not the case in the direction across the fibers. This was explained by a difference in the number and formation rate of crosslinks in the direction along the fibers and across the fibers.

### 4.5. Limitations

We would like to point out several limitations of the current study. The method for collagen fiber alignment is ideal for thin gels with thickness around 10~20 *μ*m; however, thicker gels were necessary for biaxial testing in this experiment. Thus, it might be possible that alignment of collagen fibers is not uniform in such relatively thick gel [27], which could have also caused the need to change the values of material parameters in the model. The fiber alignment method was adopted in this study for the easiness of experimental implementation. The overall research approach can be applied to other studies with more controllable alignment methods in the future. SEM was used in this study to qualitatively access the fiber alignment. Due to the higher concentration of collagen, our existing confocal microscopy capability was incapable of providing quantitative information on fiber orientation. More quantitative correlation between the structure and function of collagen scaffolds through the constitutive model might be possible by applying experimental techniques. For example, values for mean fiber orientation *γ* and dispersion around the main direction *κ* have been determined previously for collagen fibers in bladder tissue using small angle light scattering [63]. Using scanning electron microscopy techniques, fiber diameter can be quantified [61]. Fiber mechanical properties were investigated using nanoindentation [64] or micromanipulation/tensile testing of individual fibers [65].

## 5. Conclusions

Improving mechanical properties of collagen scaffolds via a controlled collagen assembly process will greatly broaden their application in numerous life science researches and will be a substantial step toward biomaterial research on tissue repair and replacement. In the present study, the mechanical properties of collagen scaffolds with the effects of crosslinking and fiber alignment were studied both experimentally and theoretically. Equibiaxial tensile tests were performed to probe the elastic properties of collagen fibrillar network within the collagen scaffolds. The stiffness of the collagen scaffolds increases with GP concentration. The anisotropic behavior of collagen scaffolds is correlated with the presence of preferred collagen fiber alignment. Our results demonstrated that crosslinking of the scaffolds can be used to tune the overall stiffness of the scaffolds without affecting the presence of anisotropy in the collagen matrix. The tangent stiffness from biaxial tensile tests is two to three orders of magnitude higher than the storage moduli from rheological measurements. This suggests that in rheological tests the movement of the interstitial fluid play a significant role in determining the overall mechanical properties of a collagen gel. Therefore, the highly hydrated collagen gel comprising stiff fibrillar network embedded in a very compliant matrix has rather low shear modulus. The stress-strain responses of both nonaligned and aligned collagen scaffolds were captured well with the anisotropic hyperelastic constitutive model. Results from simulations suggest possible changes of the mechanical property of collagen fiber in the aligned collagen scaffolds due to preferred crosslinking along the fibers. The structurally based constitutive model is promising in future studies on relating material parameters possessing physical meanings to the microstructure of collagen scaffolds.

## Figures and Tables

**Table 1:** Summary of model parameters for nonaligned and aligned collagen gels crosslinked with 0.03%, 0.1%, and 0.25% GP.

	GP (%)	*C*_10_ (kPa)	*k*_1_ (kPa)	*k*_2_	*γ* (°)	*κ*
Nonaligned	0.03	11.0	21000	600	45.0	0.333
	0.1	16.5	29750	850	45.0	0.333
	0.25	17.0	40250	1150	45.0	0.333

Aligned	0.03	11.0	5950	170	20.0	0.321
	0.1	16.5	10500	300	29.0	0.323
	0.25	17.0	11200	320	22.5	0.322
